# Serum metabolomic profiling following cerebellar repetitive transcranial magnetic stimulation combined with cognitive training on Alzheimer's disease

**DOI:** 10.3389/fneur.2026.1887127

**Published:** 2026-08-31

**Authors:** Yueqiu Chen, Jingping Shi

**Affiliations:** 1 Department of Neurology, Affiliated Nanjing Brain Hospital, Nanjing Medical University, Nanjing, China; 2 School of Basic Medical Sciences, Nanjing Medical University, Nanjing, Jiangsu, China

**Keywords:** Alzheimer's disease, cerebellum, metabolomics, mild cognitive impairment, repetitive transcranial magnetic stimulation

## Abstract

**Background:**

Complex metabolic disorders are a key characteristic of Alzheimer's disease (AD). Although studies have shown that combining repetitive transcranial magnetic stimulation (rTMS) with cognitive training (CT) can alleviate AD symptoms, the specific pathophysiological processes involved remain poorly understood. Our goal is to analyze metabolomic changes associated with bilateral cerebellar rTMS plus CT in AD treatment, to identify potential biomarkers of disease progression and therapeutic outcomes, and to validate these findings against a systematic review of existing literature.

**Methods:**

We enrolled 12 individuals with early AD and 12 healthy controls (HC). The AD group received 2 weeks of bilateral cerebellar rTMS combined with CT. We performed ultra-high-performance liquid chromatography-tandem mass spectrometry analysis on serum samples obtained from AD patients (before and after intervention) and HC. The PubMed database was used to conduct a systematic review of AD metabolomics literature, from which differential metabolites were identified and analyzed using pathway enrichment analysis.

**Results:**

A total of 510 metabolites were differentially expressed in response to treatment, with 202 consistent with improved clinical status. Fourteen pathways were enriched, including those involved in valine, leucine, and isoleucine biosynthesis, as well as glycine, serine, and threonine metabolism, which are linked to the pathology and treatment of AD. Metabolites such as lysophosphatidylcholine (22:6) and citric acid, increased after treatment and shifted toward healthy control levels, whereas γ-guanidinobutyric acid, proline, decreased after treatment. Systematic review analysis suggested Phenylalanine as a potential biomarker for AD screening at the mild cognitive impairment stage.

**Conclusion:**

This study's integrative analysis offers preliminary insights into treatment-associated metabolic alterations in AD and identifies candidate metabolites for further investigation as markers of disease status and treatment response.

**Trial registration:**

ChiCTR2200061754.

## 1 Introduction

Alzheimer's disease (AD) involves cognitive decline, along with behavioral and personality shifts. It is now well established that metabolic imbalances are a key factor in the development of AD, and the metabolic profiles of individuals with AD differ significantly from those of healthy individuals (1). As downstream products of cellular activity, metabolites indicate the physiological state of organs and tissues, including the brain. In the pathophysiology of AD, metabolomics studies have uncovered a more complex and interconnected metabolic pathway disorder beyond the classic amyloid and tau aggregates, involving multiple biological pathways and molecular mechanisms, ranging from amino acids (1) and fatty acids (1) to glucose (1), ketone bodies (by-products of neurotransmitter metabolism) (1) and a series of signaling molecules (1). Many pathophysiological mechanisms will eventually be reflected in metabolomic changes. In metabolomics research, blood samples are more widely accepted by the public than cerebrospinal fluid, making them more suitable for monitoring metabolic changes over different periods and before and after treatments (1, 2).

Currently, AD treatment mainly relies on drug therapy, along with lifestyle modifications and supportive care to manage symptoms. The effectiveness of available drugs is limited, and the safety of some medications remains a controversial concern (1). Therefore, developing innovative therapies is crucial to address the growing burden of AD. By affecting the initiation and propagation of action potentials, repetitive transcranial magnetic stimulation (rTMS) non-invasively alters the excitability and bioelectrical activity of neuronal populations. It has been used clinically in various neuropsychiatric settings and diseases (1). There are various rTMS stimulation protocols for treating AD, with early intervention primarily targeting the prefrontal cortex, but also the occipital and parietal lobes. However, the effects of single stimulation of these target areas on cognitive function in patients with AD are limited (3–5). The cerebellum, being spared in preclinical AD and only involved at the terminal stage of amyloid deposition (1), may play a key role in modulating and integrating large-scale brain network activity (5, 6) and is a popular target for neuromodulation in patients with AD in recent years (1). Our previous study confirmed that increased functional connectivity of cerebello-frontoparietal regions in the mild cognitive impairment (MCI) population may serve a compensatory role (1). Our previous clinical trials have demonstrated that cerebellar rTMS can enhance overall cognition, episodic memory, executive function, language, and visuospatial skills in patients with AD (1). In its clinical guidance, the International Federation of Clinical Neurophysiology recommends combining rTMS and cognitive training (CT) to enhance overall cognitive function (1). Several previous studies have confirmed the feasibility of combination therapy for cognitive improvement (7–9). There is evidence that rTMS may enhance the clearance of Aβ by regulating the drainage system of the brain (1), and based on previous studies, there is a multi-dimensional bidirectional regulatory pathway between peripheral metabolism and the central nervous system (1). However, the metabolic mechanism by which this regimen enhances cognitive function remains unclear.

The therapeutic target selected for this study was the cerebellum. We conducted a clinical trial combining rTMS with CT to treat early-stage AD and aimed to clarify, from a metabolomics perspective, the underlying mechanism by which the combined therapy improves cognitive function. The study used a two-pronged technical approach. First, serum samples from AD patients before and after treatment, along with healthy controls (HC), were analyzed to identify metabolites that differed between the pre- and post-treatment groups and were associated with treatment effects on the disease. Given the heterogeneity of metabolomics data, we also conducted a systematic review to validate our experimental findings against the broader literature. The differential metabolite data from previous studies were integrated, and pathway enrichment analysis was performed. By integrating experimental and literature data, this paper aims to deepen understanding of the complex pathophysiological mechanisms of AD and to identify candidate biomarkers for early screening, disease monitoring, and efficacy evaluation. The goal is to provide a theoretical basis and clinical applicability for the precise diagnosis and management of AD.

## 2 Materials and methods

### 2.1 Participants

From August 2023 to September 2024, 12 right-handed patients with AD (6 men and 6 women) were recruited from the outpatient and inpatient neurology units at Nanjing Brain Hospital, affiliated with Nanjing Medical University, China. All participants fulfilled the 2018 diagnostic criteria set by the National Institute on Aging and the Alzheimer's Disease Association Working Group (NIA-AA). Participants were eligible for the study if they were aged 50–80 years, had an mini-mental state examination (MMSE) score of 16 or higher, had at least 8 years of education, had no significant visual or hearing impairments, and, if on medication, maintained a stable dose of cholinesterase inhibitor (ChEI) or memantine for over 90 days. Exclusion criteria included a history of seizures or epilepsy; comorbid bipolar disorder, major depression, substance or alcohol abuse, severe sleep disorder, or any other neurological or psychiatric condition that could confound the study; use of barbiturates or benzodiazepines within 2 weeks prior to enrollment; and any standard contraindications for rTMS, such as metallic implants in the body or brain. Eligibility was determined by experienced study investigators, and each patient then underwent a comprehensive clinical examination and an extensive neuropsychological assessment. Simultaneously, 12 healthy individuals were recruited as controls, including 5 males and 7 females, aged 50–80, with no complaints of cognitive issues and no dementia indicated by cognitive scales. HC only underwent cognitive assessment and fasting blood sampling. All participants provided written informed consent before enrollment. The study protocol was approved by the Medical Research Ethics Committee of Nanjing Brain Hospital, China (Approval Number: 2022-KY150-01).

### 2.2 rTMS combined CT program

The rTMS procedure used a MagVenture Rapid stimulator (MagPro X100, Denmark) with a 70 mm figure-of-eight cooled coil (cool-B65; maximum output 6 T). Resting motor threshold (RMT) was determined by stimulating the motor cortex hand area to find the lowest intensity that elicited motor evoked potentials (≥50 μV in 5 of 10 pulses) from the contralateral right abductor pollicis brevis. Afterward, rTMS was administered at 100% of this threshold. During rTMS sessions, participants sat in a comfortable chair with a headrest to ensure head stability. The coil was positioned over the bilateral cerebellar Crus II using consistent scalp coordinates (1 cm below the inion and 3 cm lateral on each side) (1), and stimulation was delivered daily to both target regions. The treatment frequencies included 5, 10, and 20 Hz. The protocol involved 1 s of stimulation on each side, a 2-s interval, and 300 sets of repetitions. Each participant received 10 sessions of rTMS, once daily, over two consecutive weeks, with weekends off.

In conjunction with rTMS, AD patients completed 20 min of computer-assisted cognitive training using the “66 Brain” brain function information management platform software system. The training covered areas such as cognitive skills (including memory, attention, sensory perception, agility, executive function, and thinking abilities), language skills (including listening and comprehension, oral expression, reading, writing, object recognition, and semantic processing), and emotional and social cognition (including emotional perception, emotional understanding, and emotional regulation). The difficulty of the training was adjusted based on each subject's acceptance level, performance sre, and psychological developmental characteristics. All training tasks were selected and performed by the patients on the same touchscreen training platform, under the supervision of the same experienced therapist.

### 2.3 Assessment of clinical and neuropsychiatric status

Comprehensive and standardized neuropsychological assessments were conducted on both AD patients (before and after treatment) and HC (at enrollment). A wide range of standardized neuropsychological tests, including the MMSE, Montreal Cognitive Assessment (MoCA), Hasegawa Dementia Scale, Clinical Dementia Rating (CDR), Alzheimer's Disease Assessment Scale-Cognitive Subscale (ADAS-Cog), Rey Auditory-Verbal Learning Test (RAVLT), Clock Drawing Test (CDT), Boston Naming Test (BNT), Verbal Fluency Test (VFT), Trail Making Test Parts A and B (TMT), Symbol Digit Modalities Test (SDMT), Digit Span Test (DST), Hamilton Depression Scale (HAMD), Hamilton Anxiety Scale (HAMA), Pittsburgh Sleep Quality Index (PSQI), Activities of Daily Living (ADL) scale, Functional Activities Questionnaire (FAQ), and Rey Complex Figure Test (RCFT, including Rey-M and Rey-C), were administered to evaluate various cognitive and functional areas, such as overall cognition, episodic memory, visuospatial skills, language, executive function, attention, psychological health, sleep quality, daily activities, and visuospatial and nonverbal skills memory.

### 2.4 Experimental protocol for serum metabolic profile analysis

Morning fasting venous blood (3–5 ml) was collected into plain dry tubes, left to clot at 4 °C for 2 h, and then centrifuged at 3,000 g for 10 min. The supernatant was separated, immediately frozen at −80 °C and shipped on dry ice. Blood samples were collected under fasting conditions in the early morning (≈7:00 a.m.) on the next day after enrollment for both AD patients and health controls. For AD patients, a second fasting morning blood draw (≈7:00 a.m.) was performed on the day after the completion of rTMS therapy. Specific methods for serum ultra high-performance liquid chromatography-tandem mass spectrometry (UHPLC-MS/MS) metabolomics detection and analysis are detailed in Supplementary material 1.

### 2.5 Systematic review of metabolomics studies in AD / MCI

**Figure 1:**
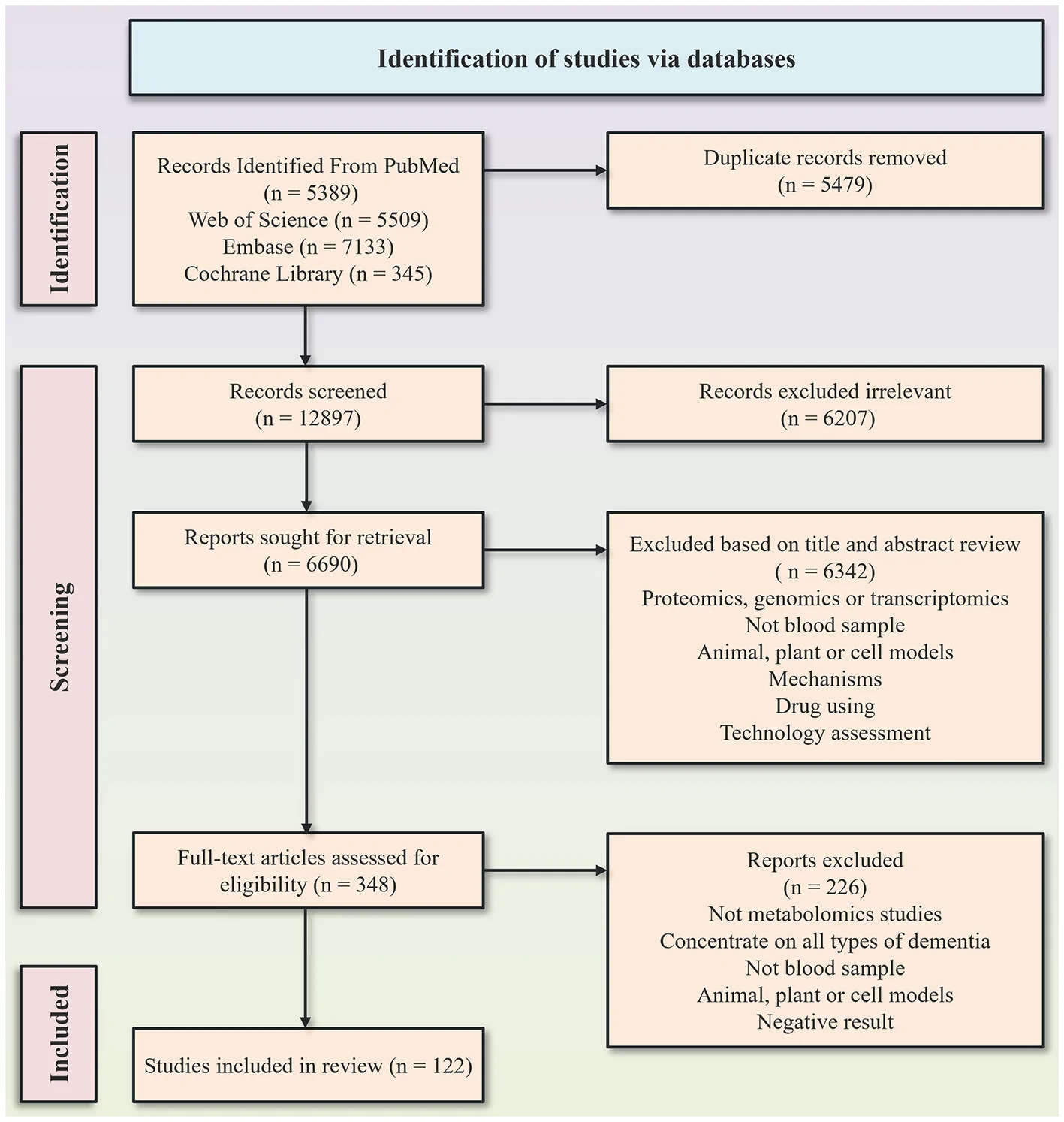
System flowchart for metabolomics research on AD/MCI. AD, alzheimer's disease; MCI, mild cognitive impairment.

The rationale for incorporating a systematic review alongside the clinical trial was to validate and contextualize the experimental findings within the broader landscape of AD and MCI metabolomics. Given the exploratory nature and small sample size of the experimental cohort, integrating literature-derived data allowed for a robust cross-reference between the treatment-related metabolic pathways identified in this study and the established disease-related pathways reported globally. This dual approach mitigates the limitations of small-cohort metabolomics, enabling a more reliable identification of candidate biomarkers and providing a stronger theoretical foundation for understanding the complex pathophysiological mechanisms underlying the combined therapy. A thorough search of the PubMed database, Web of Science, Embase and Cochrane was conducted to identify English-language metabolomics studies on Alzheimer's disease and MCI (Figure 1). The search used various combinations of keywords such as “Alzheimer disease”, “Alzheimer's disease”, “mild cognitive impairment”, “metabonomics”, “metabolomics”, “metabolite”, “lipidomics”, “GC-MS”, “LC-MS”, “Gas Chromatography-Mass Spectrometry”, and “liquid chromatography-mass spectrometry”. Review articles were excluded from the search. After removing duplicate articles and seemingly unrelated articles (such as those on other diseases) from the PubMed database, Web of Science, Embase and Cochrane in English up to **March** 15, 2026, studies meeting the following criteria were identified as consistent: (1) The study subjects were humans, with all patients meeting the diagnostic criteria for AD or MCI, and all HC confirmed to have no AD or MCI; (2) the study sample consisted of blood; (3) research used a mass spectrometry (MS) platform; (4) metabolites or pathways were listed with full names; (5) the focus was on differential metabolites or metabolic pathways related to MCI or AD progression. Exclusion criteria included: (1) studies solely involving other omics fields like genomics or proteomics; (2) studies using detection platforms such as nuclear magnetic resonance (NMR); (3) research focused on non-human models, including animals, plants, or cells; (4) samples like urine, feces, saliva, or other non-blood specimens; (5) other research types such as reviews, drug studies, mechanism investigations, clinical evaluations, and technology assessments. We performed a detailed analysis of the selected studies, extracting relevant information (e.g., publication year, author details, study design, sample type, metabolomics platform, sample size, and presence of publics) and summarizing the identified differential metabolites. Data extraction was conducted independently by two authors, Chen and Shi.

### 2.6 Statistical analysis

All statistical analyses of the clinical data were conducted using IBM SPSS 26.0 (SPSS Inc., Chicago, IL, USA). The Chi-square test was used to compare baseline characteristics between the two groups. Quantitative variables were first checked for normal distribution and equality of variances. Shapiro-Wilk was used to test for normality, and Levene's test was used to assess variance equality. For continuous variables that met both criteria, an independent-samples *t*-test was performed; otherwise, the Wilcoxon rank-sum test was used. To analyze the change in scale scores before and after treatment in the case group, the difference in scores was calculated. For differences that met the normality assumption, a paired *t*-test was used; for non-normal differences and ordinal data, the Wilcoxon signed-rank test was used. In all analyses, a two-tailed *p* value below the specified threshold of 0.05 was considered statistically significant.

R (version: 4.0.3) and the R package were used for all multivariate data analysis and modeling. The Pareto proportion method was used for mean-centered analysis of the data. The model was established based on principal component analysis (PCA), orthogonal partial least squares discriminant analysis (PLS-DA) and partial least squares discriminant analysis (OPLS-DA). All models evaluated were tested for overfitting using the permutation test method. The descriptive performance of the model was determined by R2X (cumulative) [perfect model: R2X (cum) = 1] and R2Y (cumulative) [perfect model: R2Y (cum) = 1] values, and the predictive performance was measured by Q2 (cumulative) [perfect model: Q2 (cum) = 1] and permutation test (n = 200). The permutation model does not predict the category: the R2 and Q2 values at the y-intercept must be lower than those of the non-permutation model. OPLS-DA allows the use of variable projected importance (VIP) to determine the differential metabolites. The VIP score value represents the contribution of a variable to the discrimination of all category samples. Mathematically, these scores were calculated as the sum of squares of the PLS weights for each variable. The mean VIP value was 1, and usually a VIP value >1 was considered significant. Higher scores are consistent with greater discrimination and thus constitute criteria for biomarker selection. At the univariate analysis level, the threshold for statistical significance of VIP values obtained in the OPLS-DA model and a two-tailed Student's *t*-test (*p* value) for standardized raw data were used to obtain the discriminating metabolites. One-way analysis of variance (ANOVA) was used to calculate *p* values for multi-group analysis. Metabolites with VIP values >1.0 and *p* values < 0.05 were considered statistically significant. The fold change was calculated as the log of the average mass response (area) ratio between any two categories. On the other hand, cluster analysis of the identified differential metabolites was performed using the R package.

Kyoto encyclopedia of genes and genomes (KEGG) enrichment analysis of the identified differential metabolites was conducted using Fisher's exact test, with a significance threshold of *p* < 0.05. Pathway annotation and visualization were facilitated by the KEGG database and MetaboAnalyst 6.0, respectively (http://www.metaboanalyst.ca/).

Given the exploratory nature of this study, no adjustment for multiple comparisons was performed in the metabolomics analyses.

## 3 Results

### 3.1 Clinical characteristics

A total of 12 AD patients (6 males, 6 females) and 12 HC (5 males, 7 females) were recruited. The two groups were comparable with respect to gender, age, and educational level. The comparison of scores on each scale between the two groups is shown in Table 1. All subjects completed 10 sessions of rTMS treatment and cognitive training, along with pre- and post-treatment scale assessments and blood sampling. All controls completed scale assessments and fasting blood sampling. Because some subjects did not complete the TMT (B) and SDMT scales, those scales were not included in the analysis.

**Table 1:** Demographic and clinical characteristics of the subjects at baseline.

Characteristics	Patient	Control	*p* value
	n = 12	n = 12	
Gender (male/female)	6/6	5/7	1.000
Age (years)	68.50 [58.50, 73.00]	63.50 [61.25, 65.50]	0.271
edu-t (years)	10.50 [6.75, 14.25]	9.00 [6.75, 12.00]	0.699
MMSE	18.50 [17.25, 21.75]	28.50 [28.00, 29.00]	< 0.001
MOCA	12.00 [11.00, 14.75]	26.00 [24.25, 27.00]	< 0.001
HDS	20.75 [14.88, 25.88]	31.50 [29.75, 32.50]	< 0.001
CDR	0.50 [0.50, 1.00]	0.00 [0.00, 0.00]	< 0.001
HAMD	3.58 ± 2.476	6.67 ± 2.23	0.004
HAMA	4.08 ± 1.51	4.83 ± 2.17	0.336
PSQI	3.08 ± 1.83	8.50 ± 4.03	0.001
ADL	22.00 [20.00, 24.75]	20.00 [20.00, 21.75]	0.041
FAQ	3.00 [0.25, 5.50]	0.50 [0.00, 1.00]	0.024
AVLT	15.00 [12.25, 16.75]	4.00 [4.00, 5.75]	< 0.001
REY-m	0.00 [0.00, 0.00]	18.00 [12.75, 22.75]	< 0.001
REY-c	32.50 [28.38, 34.88]	36.00 [34.13, 36.00]	0.016
CDT1	2.00 [1.00, 4.00]	4.00 [2.50, 4.00]	0.050
CDT2	14.50 [4.50, 21.00]	22.00 [13.25, 24.00]	0.030
DST	6.50 [5.25, 10.00]	10.50 [8.25, 11.75]	0.029
BNT	19.00 [15.50, 21.75]	27.00 [23.75, 28.00]	0.001
VFT	9.50 ± 2.81	18.00 ± 4.26	< 0.001
TMT(A)	107.67 ± 34.47	54.92 ± 11.94	< 0.001

MMSE, mini-mental state examination; MoCA, montreal cognitive assessment; HDS, hasegava dementia scale; CDR, clinical dementia rating; HAMD, hamilton depression scale; HAMA, hamilton anxiety scale; PSQI, pittsburgh sleep quality index; ADL, activities of daily living; FAQ, functional activities questionnaire; AVLT, auditory-verbal learning test; CDT, clock drawing test; DST, digit span test; BNT, boston naming test; VFT, verbal fluency test; TMT, trail making test.

### 3.2 Neuropsychological assessment

Following the combined intervention, significant improvements were observed in several cognitive domains. Specifically, in the domain of overall cognition, MoCA (*p* = 0.005) and HDS (*p* = 0.022) scores increased significantly. In terms of language function, BNT scores showed significant improvement (*p* = 0.021). For visuospatial and nonverbal memory, Rey-M scores also increased significantly (*p* = 0.028). Additionally, dementia severity, as measured by the CDR, decreased significantly (*p* = 0.025, *Z* = −2.236). No significant changes were observed in the remaining scales assessing episodic memory, executive function, attention, psychological health, sleep quality, or daily activities. All details were shown in Table 2.

**Table 2:** Comparison of scale scores between subjects before and after combined treatment.

Scale	Before	After	*p* value
MMSE	19.42 ± 2.353	20.75 ± 3.841	0.164
MOCA	12 [11, 14.75]	16 [11.25, 18.75]	0.005
ADAS	20.7975 ± 5.87709	17.7983 ± 5.26437	0.088
HDS	20.375 ± 5.4819	23.833 ± 5.2151	0.022
CDR	0.5 [0.5, 1]	0.5 [0.5, 0.5]	0.025
HAMD	3.58 ± 2.466	2.33 ± 1.614	0.124
HAMA	4 [5, 5]	3 [2, 5]	0.392
PSQI	3.08 ± 1.832	3 ± 2.132	0.886
ADL	22.42 ± 2.314	21.42 ± 1.564	0.089
FAQ	3.17 ± 3.01	3.5 ± 3.205	0.704
AVLT	14.5 ± 2.714	16.25 ± 3.334	0.108
REY-m	0 [0, 0]	0.5 [0, 6.25]	0.028
REY-c	31.667 ± 3.7558	31.375 ± 3.6626	0.794
CDT1	2.17 ± 1.586	2.33 ± 1.497	0.767
CDT2	13.33 ± 8.521	14.75 ± 7.047	0.615
DST	7.67 ± 2.995	8.5 ± 2.355	0.117
BNT	19.17 ± 3.857	21 ± 3.861	0.021
VFT	9.5 ± 2.812	9.58 ± 5.035	0.941
STT(A)	107.67 ± 34.466	100.58 ± 39.759	0.345

MMSE, mini-mental state examination; MOCA, montreal cognitive assessment; HDS, hasegava dementia scale; CDR, clinical dementia rating; HAMD, hamilton depression scale; HAMA, hamilton anxiety scale; PSQI, pittsburgh sleep quality index; ADL, activities of daily living; FAQ, functional activities questionnaire; AVLT, auditory-verbal learning test; CDT, clock drawing test; DST, digit span test; BNT, boston naming test; VFT, verbal fluency test; TMT, trail making test.

### 3.3 Serum metabolic profile analysis

#### 3.3.1 Quality assessment of metabolomics platform

**Figure 2:**
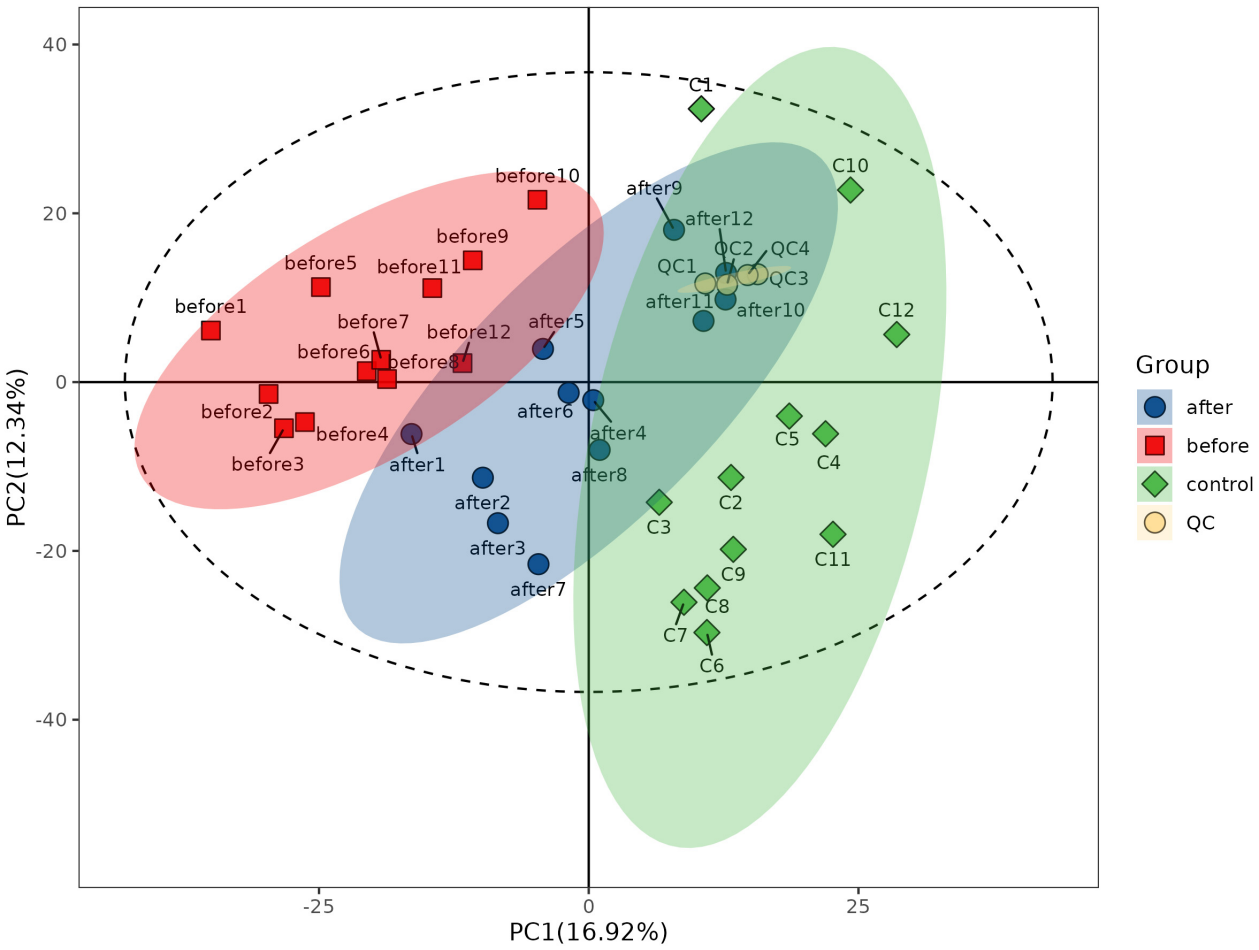
Score plot of PCA analysis for the overall sample of combined positive and negative ion patterns. PCA, principal component analysis.

After data normalization, PCA of peak intensities from all study and quality control (QC) samples were conducted. The QC samples formed a tight cluster in the mixed ionization mode, indicating strong experimental reproducibility (Figure 2). Pearson correlation coefficients among QC samples were all above 0.9, confirming high inter-sample correlation (Supplementary material 6). Hotelling's *T*^2^ test was used to identify outlier samples in the combined dataset, which included positive- and negative-ion patterns. Using a 95% confidence interval (blue dashed line) and a 99% confidence interval (red dashed line) as criteria for outlier detection, the *T*^2^ value of each group was below the threshold for the 95% confidence interval. All samples fell within the 95% and 99% confidence intervals, with no outliers detected, demonstrating good consistency and stability across the dataset (Supplementary material 7).

#### 3.3.2 Differential metabolite identification and pathway enrichment analysis

**Figure 3:**
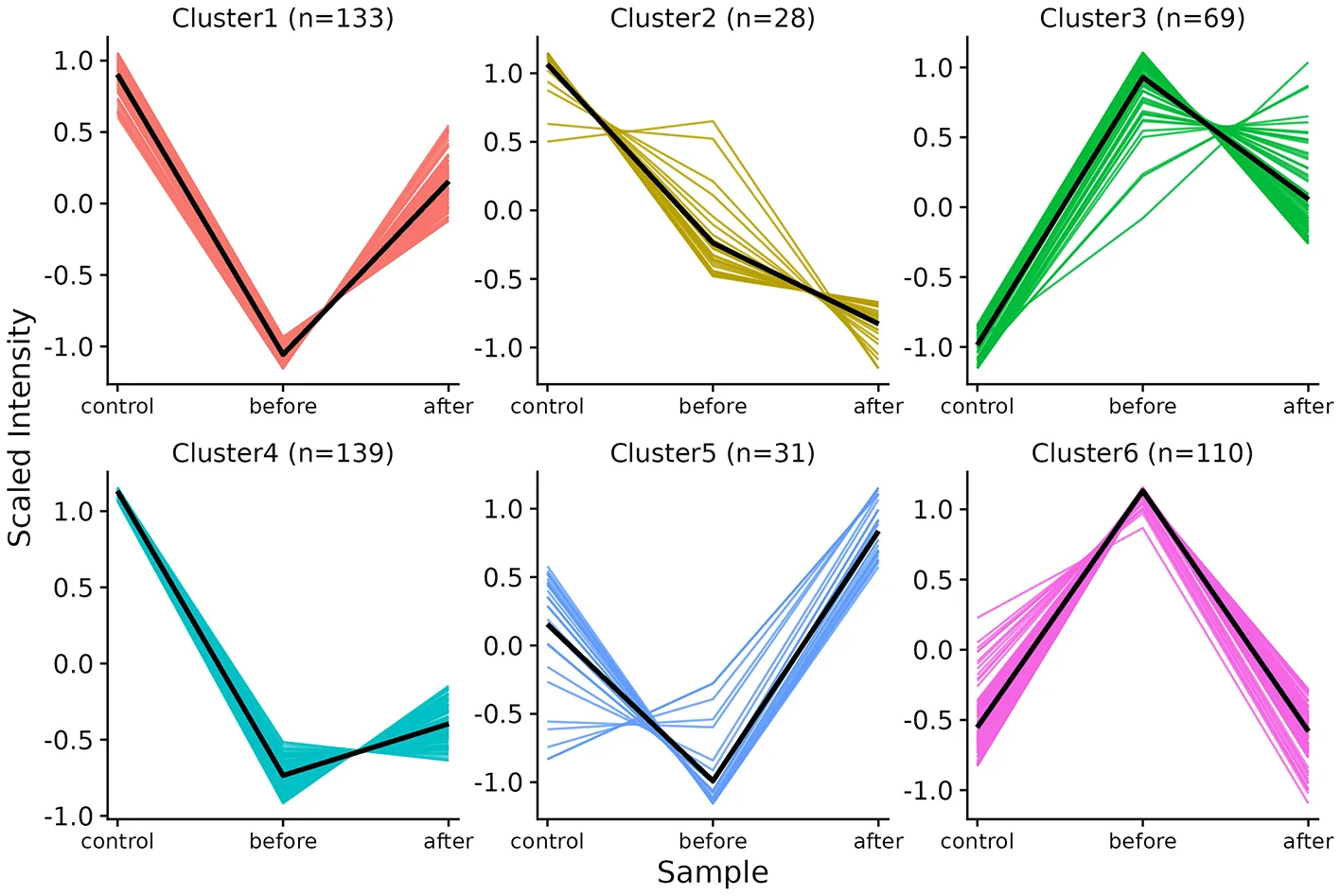
K-means clustering trend plot. The *y*-axis represents the expression level of metabolites after *Z*-score normalization of the group means mass spectrometry intensity.

With *p* < 0.05 and PLS-DA VIP > 1 as cutoff point, 510 metabolites were identified as differentially abundant. The K-means clustering algorithm was used to group the metabolites into several clusters, with the number of metabolites in each cluster indicated above it. For each cluster, the *x*-axis represents different groups, and the y-axis shows the relative expression level. Each line shows how the expression of a specific metabolite changes across groups. As shown in Figure 3, the black line indicates the trend of the average expression levels of all metabolites within the cluster across the different groups. We selected metabolites that exhibited significant baseline deviations from healthy controls and showed partial normalization after treatment. Cluster 2 was excluded as its deviation worsened post-treatment. Cluster 4 was excluded due to minimal reversal. Clusters 5 and 6 were also excluded because their post-treatment levels overshot the normal range instead of showing partial recovery. Ultimately, clusters 1 and 3 were retained for further investigation, involving 202 metabolites in total. Cluster 1 metabolites increased and cluster 3 metabolites decreased after treatment. The detailed substances are listed in Supplementary material 2.

A total of 63 metabolic pathways were enriched, of which 14 showed significant alterations (*p* < 0.05). These significantly affected pathways, primarily involving amino acid metabolism, protein synthesis, small-molecule metabolism and biosynthesis, substrate transport, and overall metabolic regulation. The specific pathways are listed in Table 3.

**Table 3:** Metabolic pathways associated with combination therapy in AD.

Pathways' name	Total no.	Hits no.	*p*-value
Protein digestion and absorption	47	6	< 0.001
Biosynthesis of amino acids	128	7	0.001
D-Amino acid metabolism	69	5	0.002
Glycine, serine and threonine metabolism	48	4	0.003
beta-Alanine metabolism	32	3	0.007
ABC transporters	138	6	0.007
Metabolic pathways	3,048	46	0.018
Histidine metabolism	47	3	0.021
Lysine degradation	51	3	0.026
Aminoacyl-tRNA biosynthesis	52	3	0.027
Valine, leucine and isoleucine biosynthesis	23	2	0.033
Porphyrin metabolism	148	5	0.038
Pyrimidine metabolism	64	3	0.046
Glycosylphosphatidylinositol (GPI)-anchor biosynthesis	4	1	0.0496

AD, alzheimer's disease.

### 3.4 A systematic review of metabolomics studies on AD and MCI

#### 3.4.1 Overview of eligible studies

The PubMed database, Web of Science, Embase and Cochrane literature search identified 18,376 articles. After screening by title, abstract, and keywords, removing duplicates and reviews, and excluding articles unrelated to the research topic (e.g., animal studies, other diseases, different samples, mechanism studies, drug use, technology evaluation), 348 records remained. Following a thorough full-text review, 122 articles met the inclusion criteria. Supplementary material 3 provides an overview of the included studies, summarizing their design, cohort size, sample origin, sample type, metabolomics platform. The risk assessment can be found in Supplementary material 3. The numbers of AD cases per study ranged from 11 to 1,428, MCI cases from 10 to 491, and HC from 5 to 1,796. Among metabolomics platforms, LC–MS was used in 74 studies, GC–MS in 36, and other methods, such as capillary electrophoresis-mass spectrometry (CE–MS), direct-injection mass spectrometry, and additional approaches were also reported. 54 of these studies used plasma, 43 used serum, and 7 used whole blood or both. Of these studies, 95 were case-control, 13 were cohort, 9 were cross-sectional, 3 were comprehensive analyses, and 2 used Mendelian randomization. A total of 23 studies used public databases either wholly or in part.

#### 3.4.2 Metabolites associated with AD/MCI

In the comparison between AD and HC, with 322 occurring more than once; see Supplementary material 4. Valine and tryptophan were the most frequently observed metabolite, appearing 13 times, followed by taurine, and lysophosphatidylcholine (LPC) (18:1), each with 10 mentions. Histidine, glutamic acid and LPC (16:0) appeared 9 times, while SM (d18:1/16:0) and LPC (18:0), each appeared 8 times. Additionally, 630 metabolites showed significant differences between MCI and HC in system review, with 58 appearing multiple times (Supplementary material 4). Phenylalanine was the most frequently observed metabolite, appearing six times, followed by valine, glutamic acid, and LPC (18:1), each appearing four times. In the comparison between AD and MCI, 152 metabolites differed. Among these, nine metabolites (tryptophan, threonine, serine, proline, methionine, glycine, glutamic acid, LPC [16:0], and LPC [16:1]) occurred twice across the dataset.

#### 3.4.3 Metabolic pathways associated with AD/MCI

**Figure 4:**
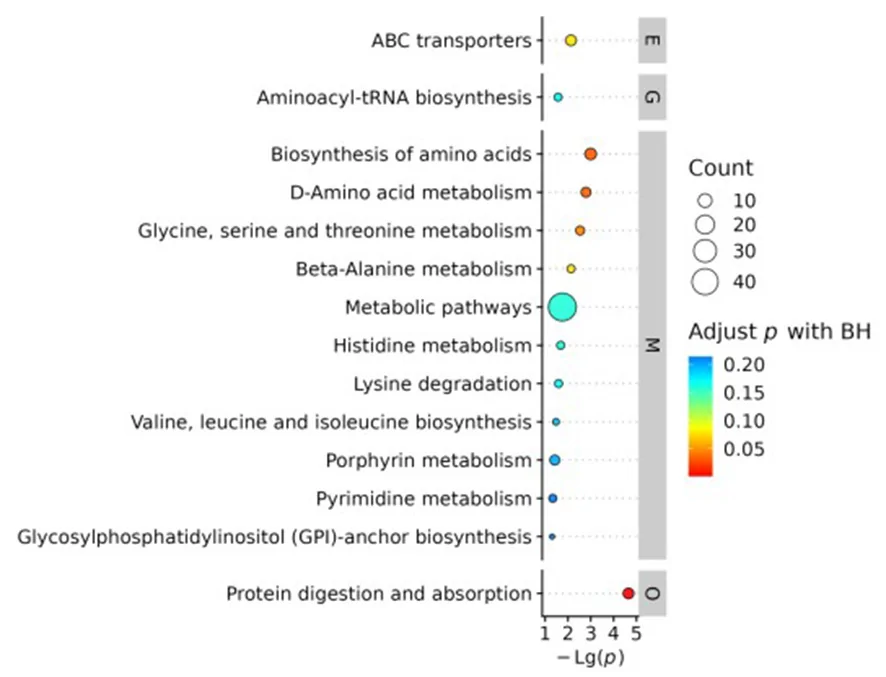
KEGG pathway enrichment bubble plot for the control, before-treatment, and after-treatment comparison groups. Each bubble represents an enriched KEGG pathway. The x-axis shows the enrichment significance as –log10(*p*), with larger values indicating stronger enrichment. Bubble size represents the number of differential metabolites mapped to each pathway, and bubble color indicates the Benjamini–Hochberg-adjusted *p* value, with warmer colors representing greater statistical significance.

In the comparison between AD and HC, 63 pathways differed, of which 21 were statistically significant (*p* < 0.05). For MCI vs. HC, 54 pathways showed alterations, including 13 with *p* < 0.05. In comparison of AD vs. MCI, 41 pathways were affected, 11 of which were significantly enriched (*p* < 0.05). Figure 4 illustrates that KEGG enrichment analysis identified 13 pathways among the control, before-treatment, and after-treatment groups. Protein digestion and absorption showed the strongest enrichment, while metabolic pathways contained the largest number of differential metabolites. Several amino acid-related pathways, including amino acid biosynthesis, D-amino acid metabolism, glycine, serine and threonine metabolism, and beta-alanine metabolism, were also prominently enriched.

#### 3.4.4 Intersection analysis of metabolic pathways

**Figure 5:**
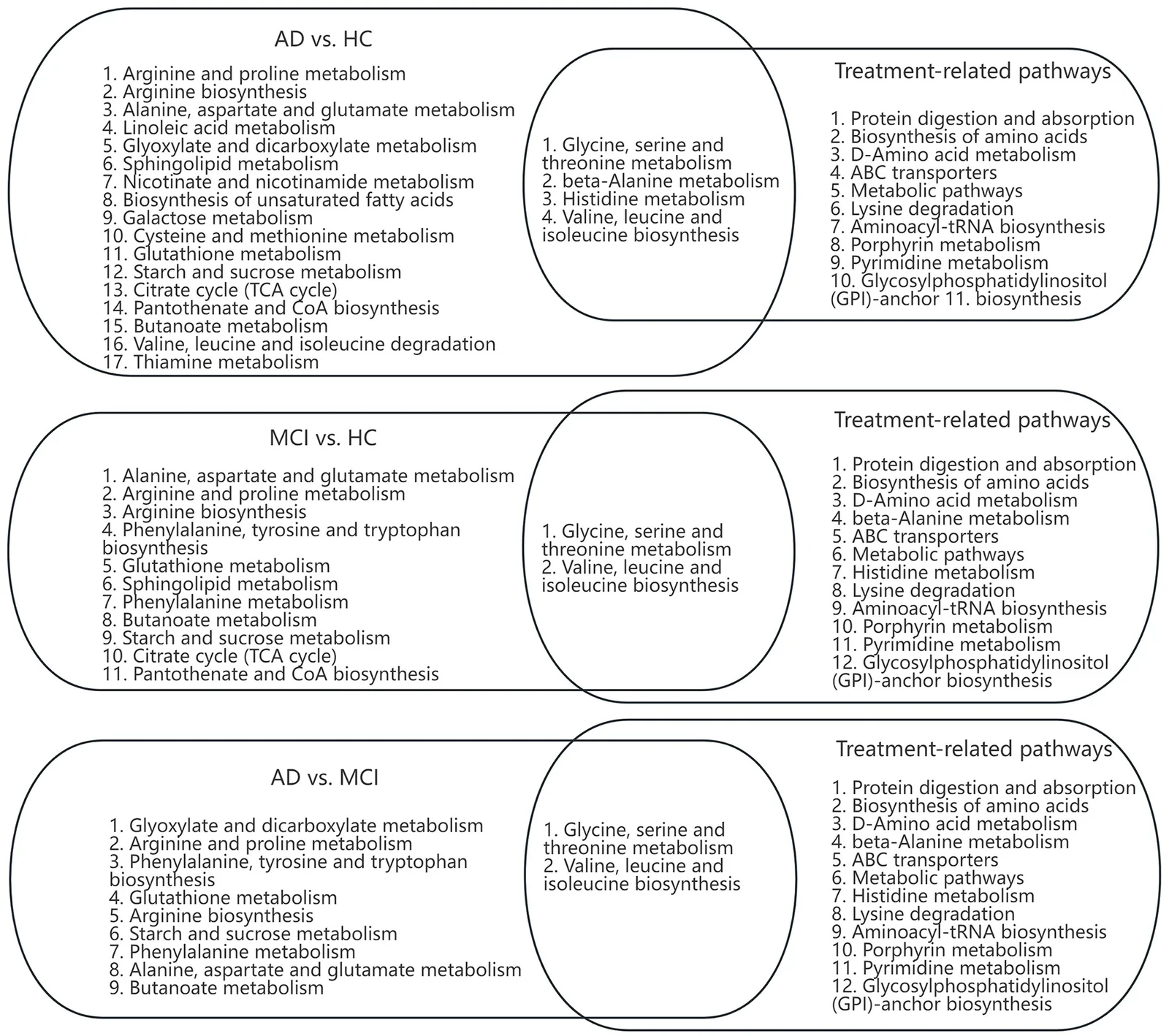
Pathway intersection analysis between AD and MCI and those associated with combination therapy. AD, alzheimer's disease; MCI, mild cognitive impairment; HC, healthy controls; TCA, tricarboxylic acid cycle.

We compared this study with the pathways derived from this systematic review for AD and MCI. Figure 5 shows that the intersection of treatment-related pathways and AD-related pathways includes four amino acid metabolic pathways: glycine, serine and threonine metabolism; β-alanine metabolism; histidine metabolism; and the biosynthesis of valine, leucine, and isoleucine. The overlap between this study and McI-related pathways involves glycine, serine, and threonine metabolism, as well as the biosynthesis of valine, leucine, and isoleucine, which are also shared by previous AD and MCI-related studies.

#### 3.4.5 Metabolic markers associated with AD treatment

**Figure 6:**
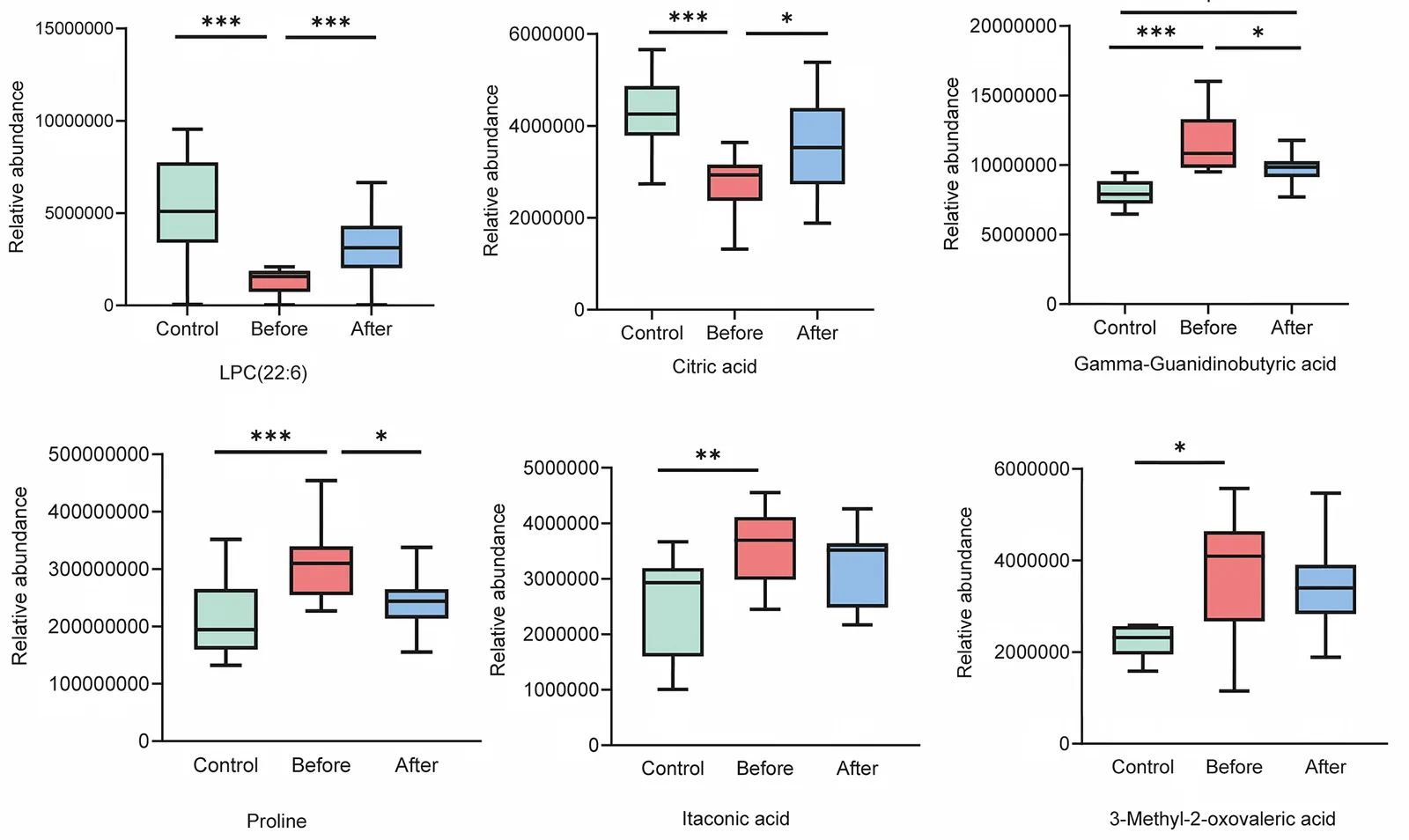
Comparison of AD- and treatment associated with metabolites makers. *P ≤ 0.05 (significant); **P ≤ 0.01 (highly significant/extremely significant); ***P ≤ 0.001 (extremely highly significant).

We compared the metabolites of clusters 1 and 3 in this study with those reported in previous studies and with those of AD and MCI. After removing exogenous substances, six substances were identified as associated with both disease and treatment (Figure 6). These included LPC (22:6), citric acid, γ-guanidino butyric acid, proline, itaconic acid, and 3-methyl-2-oxovaleric acid.

Among them, LPC (22:6) and citric acid belong to cluster 1, which are increased after treatment, moving closer to healthy control levels. Regarding LPC (22:6), previous studies in our systematic review consistently reported decreased levels in both AD vs. HC and MCI vs. HC comparisons (10–12), consistent with our findings. For citric acid, previous studies reported decreased levels in MCI vs. HC comparisons (13, 14), which aligns with our results; however, in AD vs. HC comparisons, one study reported elevated levels (15), while another did not specify the direction of change (16).

γ-Guanidino butyric acid, proline, itaconic acid, and 3-methyl-2-oxovaleric acid belong to cluster 3. Their altered may reflect disease-associated metabolic alterations. For proline, previous studies reported decreased levels in AD vs. HC comparisons (14, 17), or did not specify the direction (18); in AD vs. MCI comparisons, both decreased (14) and increased (19) levels were reported. Notably, these previous findings are inconsistent with our observation of elevated proline levels in AD patients compared to HC.

For itaconic acid, previous studies did not specify the direction of change in AD vs. HC comparisons (16), while decreased levels were reported in MCI vs. HC comparisons (20), which is inconsistent with our findings. For 3-methyl-2-oxovaleric acid, previous studies reported decreased levels in AD vs. HC (17, 19) or did not specify the direction of change (16, 21).

## 4 Discussion

To date, only a limited number of studies have investigated the metabolic effects of rTMS in AD patients. Velioglu et al. (22) reported that rTMS significantly increased hippocampal N-acetylaspartate (NAA) levels in AD patients, as measured by magnetic resonance spectroscopy, and that this increase was associated with improved visual memory performance. Similarly, Zhang et al. found that high-frequency rTMS combined with cognitive training significantly elevated the NAA/creatine (NAA/Cr) ratio in the left dorsolateral prefrontal cortex of patients with mild-to-moderate AD. Moreover, changes in NAA/Cr ratio were negatively correlated with changes in ADAS-Cog scores, indicating that greater increases in NAA/Cr were associated with greater cognitive improvement (23). These prior studies primarily focused on NAA—a marker of neuronal integrity and mitochondrial function—suggesting that rTMS-based interventions may enhance neuronal viability and energy metabolism in specific brain regions. Notably, to the best of our knowledge, no previous study has systematically evaluated the effects of rTMS combined with cognitive training on serum metabolomic profiles in AD patients using untargeted or targeted metabolomics approaches. Although the aforementioned MRS-based studies provided valuable insights into localized metabolic changes (e.g., NAA), particularly changes in NAA within specific brain regions, they did not capture the broader systemic metabolic alterations that may occur in response to the intervention.

The bilateral cerebellar rTMS, combined with the cognitive training protocol used in this study, demonstrates high adherence and safety, post-intervention scores showed improvement. A well-established bidirectional connection exists between the cerebellum and the cerebral cortex, as shown in previous research. Signals from the cerebral cortex, basal ganglia, and limbic regions are transmitted to the cerebellum via the anterior pontine nucleus, and the cerebellum sends feedback signals to telencephalic areas such as the motor and prefrontal cortices via the deep cerebellar nucleus, red nucleus, and anterior thalamic nucleus. These bidirectional connections, supported by anatomical and functional data, play a key role in regulating cognitive function (6, 24). Metabolomics studies have revealed that patients with cognitive impairment exhibit disorders of neurotransmitter metabolism, abnormal energy metabolism, and heightened cerebellar inflammatory responses (6, 25–28). Previous studies have suggested that rTMS may improve the permeability of blood-brain barrier, accelerate the clearance of Aβ deposition (29), and may improve oxidative stress balance and neurotrophic support. These changes may be related to the effects of rTMS on improving cognitive function and inhibiting pathological damage in the brain of AD patients (29). These issues may directly affect signal transduction within cognitive pathways. The primary goal of cognitive training is to facilitate compensation and remodeling of cognitive functions through targeted task reinforcement (29). However, the effect of cognitive training alone may be limited by the decline in cortical neural plasticity in patients with AD. Bilateral cerebellar rTMS may modulate the identified pathways to enhance neural responsiveness to cognitive training, thereby amplifying its effect. This rationale is supported by the fact that cognitive impairment in Alzheimer's disease often appears with bilateral symmetry. Bilateral cerebellar rTMS can equalize cognitive functions between the left and right cerebral hemispheres by symmetrically stimulating both cerebellar hemispheres, preventing the functional imbalance caused by unilateral stimulation.

A systematic review of previous studies found that amino acids such as valine, taurine, tryptophan, histidine, glutamic acid, and those from the LPC family often appeared in comparisons between AD and HC. An imbalance in branched-chain amino acid biosynthesis and metabolism could be a common metabolic hallmark of AD (29), potentially leading to disturbances in neuronal energy supply, insulin resistance, increased Aβ deposition, and tau protein abnormalities phosphorylation. The high frequency of the LPC family indicates that LPC metabolic disorder is not limited to specific subtypes, and that various LPC subtypes may contribute to AD pathology by regulating lipid metabolism, synaptic repair, inflammatory responses, and other processes (29). In the comparison between MCI and HC, phenylalanine was frequently detected and emerged as a key metabolite for distinguishing between the two groups. Phenylalanine is an aromatic amino acid, and its abnormal metabolism can lead to disorders of neurotransmitter synthesis (e.g., dopamine, norepinephrine) and trigger oxidative stress and brain inflammation (29). MCI, as a prophase of AD, exhibits metabolic disturbances that have not yet reached the severity seen in AD. Our systematic review shows that the significant difference in phenylalanine levels suggests that it may serve as a biomarker for early MCI screening. Such metabolomic signatures could aid in early AD diagnosis and treatment planning. Additionally, the higher frequencies of valine and glutamic acid in MCI compared to HC suggest that disorders in branched-chain amino acid metabolism and excitatory neurotransmitters may begin during the MCI stage.

Among the substances we identified as being associated with both disease status and treatment response, LPC (22:6) and citrate exhibited increased levels after treatment, Conversely, γ-guanidinobutyric acid, proline, itaconic acid, showed decreased levels after treatment. LPC (22:6) serves as the transport form of docosahexaenoic acid (DHA), which is the most abundant polyunsaturated fatty acid in the brain. As a major component of neuronal synaptic membranes, DHA is essential for synaptic plasticity and cognitive function. DHA levels are significantly decreased in the brains of AD patients, leading to impaired synaptic function (30). In the present study, LPC (22:6) levels increased after combined treatment, suggesting that the treatment may enhance its synthesis and release, increase DHA availability in the brain, and aid in repairing synaptic function. A recent large-scale plasma metabolomics study demonstrated that lysophosphatidylcholines (lysoPCs), particularly those carrying polyunsaturated fatty acids, were negatively associated with plasma P-tau 181 levels and biomarker-supported Alzheimer's disease (31). Moreover, LPC metabolic modules have been identified as significantly associated with cross-sectional amyloid/tau biomarkers in the Alzheimer's Disease Neuroimaging Initiative (ADNI) cohort (Circulating lipids are related to longitudinal changes of ATN biomarkers for Alzheimer's disease) (32), suggesting that alterations in LPC metabolism, including LPC (22:6), may be related to Aβ and phosphorylated tau pathology and may reflect a potentially favorable metabolic profile. Citric acid is a crucial component of the tricarboxylic acid cycle, linking energy production to the regulation of oxidative stress. AD patients experience mitochondrial dysfunction and tricarboxylic acid cycle disorders, which result in energy deficiency, reactive oxygen species buildup, and neuronal damage (1). Following combined treatment, citric acid levels increased, which may activate the tricarboxylic acid cycle, enhance mitochondrial function, reduce oxidative stress, and provide neuroprotection. Critically, citrate has been shown to inhibit Aβ 25–35 and Aβ 1–40 aggregation *in vitro* (33), and mitochondrial dysfunction is known to affect both Aβ aggregation and tau hyperphosphorylation (34). Thus, the treatment-related increase in citrate may reflect improved mitochondrial energy metabolism, potentially counteracting these pathogenic processes. γ-Guanidinylbutyric acid is an abnormal product of arginine metabolism that can inhibit neuronal energy metabolism and induce apoptosis (30). It has been shown that Aβ oligomers impair GABAergic inhibitory synaptic transmission, leading to neuronal network hyperexcitability, which in turn creates a vicious cycle that promotes further Aβ deposition and tau pathology (35). Proline buildup can worsen oxidative stress and inflammation (30). Beyond its effects on oxidative stress and inflammation, proline metabolism has been linked to AD core pathology through the PREPL pathway, as downregulation of PREPL has been shown to increase Aβ 42 secretion and Tau phosphorylation (36). Itaconic acid is a byproduct of macrophage metabolism, and elevated levels can amplify inflammatory responses (30). 3-methyl-2-oxovaleric acid is an intermediate in leucine metabolism, and its buildup can disrupt neuronal energy metabolism (30). In line with this, a CSF metabolomics study demonstrated that 3-methyl-2-oxovalerate levels were significantly elevated in AD dementia patients and showed the highest anticorrelations with Aβ42/p-tau and Aβ42/t-tau ratios (37). In the present study, we found the combined therapy significantly altered the levels of these metabolites, potentially reflecting treatment-related modulation of metabolic processes associated with AD. Importantly, these metabolites should be considered candidates for further investigation rather than established biomarkers or therapeutic targets, and their clinical relevance requires confirmation in independent validation studies.

Combined with previous studies on AD/MCI metabolomics, glycine-serine-threonine metabolism, β-alanine metabolism, histidine metabolism, and branched-chain amino acid (BCAA) biosynthesis, these pathways are the main pathways involved in the progression of AD pathology and in the response to combined therapy. Their abnormal regulation is closely linked to the neuropathological mechanisms of AD. Glycine, serine, and threonine metabolism form a major amino acid metabolic pathway in the human body, participating in various physiological processes, including one-carbon metabolism, neurotransmitter synthesis, and antioxidative functions under stress (30). Glycine and serine form a closely interconnected regulatory network with the glutamate system, receptor co-activation, and metabolic pathways, regulating excitability and synaptic plasticity in the central nervous system. Disruptions in this network in AD (such as decreases in D-serine/glycine and glutamate excitotoxicity) significantly contribute to cognitive decline impairment (30). Combined treatment may restore metabolic levels through this pathway, promoting neuronal repair via one-carbon metabolism and stabilizing cerebral cortex excitability by balancing neurotransmitters, thereby enhancing cognition (30). β-alanine acts as a precursor for the inhibitory neurotransmitter gamma-aminobutyric acid (GABA), and disruptions in its metabolic pathway directly reduce GABA synthesis. In Alzheimer's disease brains, the substantial loss of GABAergic neurons disrupts the excitatory–inhibitory balance of neural activity circuits (30). The combined treatment could activate the β-alanine metabolic pathway, increase GABA production and release, and repair damaged neural circuits. Histamine, a key product of histidine metabolism, is an essential neuromodulator that regulates cognitive functions, sleep-wake cycles, and other physiological processes. In patients with AD, the loss of histaminergic neurons and the associated decrease in cerebral histamine levels result in reduced cognitive flexibility and impairments in encoding and retrieval (30). Regulating this pathway with combination therapy may enhance histamine synthesis and suppress inflammatory responses in the brain, thereby improving cognitive function. BCAAs are crucial energy sources involved in protein synthesis and neurotransmitter metabolism. An imbalance in BCAA metabolism in the peripheral blood and brain of patients with AD not only results in insufficient energy and materials for neurons and damages synaptic function but also induces insulin resistance, which further exacerbates Aβ deposition and tau protein phosphorylation (1, 38). Combining therapy may restore BCAAs‘ metabolic balance, reduce insulin resistance, and slow the progression of AD by increasing BCAAs' biosynthesis.

From the perspective of disease progression, the glycine-serine-threonine and BCAA biosynthesis pathways appear to represent core metabolic disturbances inherent to AD pathophysiology, with their dysregulation detectable across multiple stages of the disease (39, 40). The progressive recruitment of β-alanine and histidine metabolism may indicate a shift toward more advanced stages involving widespread neurotransmitter system failure, as GABAergic and histaminergic systems become progressively compromised with advancing disease (41). This sequential pathway involvement may serve as a metabolic signature of AD progression. Regarding treatment response, the glycine-serine-threonine and BCAA pathways, being central to AD pathophysiology, may represent primary targets for disease-modifying interventions. Notably, restricting individual BCAAs—particularly isoleucine or valine—has been shown to improve short-term memory in mouse models of AD, with sex-specific effects (42), suggesting that BCAA-targeted dietary interventions could form the basis of novel therapeutic strategies. The β-alanine pathway, through its modulation of GABAergic and glutamatergic neurotransmission, may offer a target for symptomatic cognitive improvement (41). Histidine metabolism, via its role in histamine synthesis, may be targeted to enhance cognitive function and suppress neuroinflammation.

This is an exploratory study; although some results have been obtained, the sample size is small and long-term follow-up is lacking, making it difficult to verify the long-term effectiveness of combined therapy and to assess the dynamic changes in metabolic indicators. Also, the lack of a sham control group makes it difficult to determine the precise effect of rTMS. Results from non-targeted metabolomics require targeted quantitative detection in a large sample cohort to confirm the sensitivity and specificity of metabolic markers. The significant heterogeneity among the studies included in this systematic review requires large-sample verification of the reproducibility of frequently reported metabolites and pathways. Future studies should increase sample sizes, conduct long-term follow-up, incorporate multi-omics approaches to build regulatory networks, analyze mechanisms of action, and implement targeted intervention experiments.

## 5 Conclusion

This study suggests that cerebellar-targeted rTMS combined with cognitive training may improve multiple cognitive domains in patients with AD. By integrating metabolomics detection with a systematic review, this study provides preliminary insights into the potential metabolic mechanisms underlying this combined intervention and highlights several candidate metabolites potentially associated with disease status or treatment response. LPC (22:6) and citric acid showed changes associated with favorable treatment-related metabolic effects, whereas γ-guanidinobutyric acid, proline, indicated changes that may be associated with unfavorable disease-related metabolic processes. Phe was identified as a potential candidate biomarker for AD screening at the MCI stage. This study offers preliminary clinical and metabolomic evidence regarding the potential mechanism underlying the combined use of cerebellar rTMS and cognitive training in AD. These findings may provide important support for future precision treatments, biomarker development, and early intervention in AD.

## Data Availability

The original contributions presented in the study are included in the article/Supplementary material, further inquiries can be directed to the corresponding author.
