# Supplementary material for: Serum metabolomic profiling following cerebellar repetitive transcranial magnetic stimulation combined with cognitive training on Alzheimer's disease
Source: Front Neurol. 2026 Aug 31;17:1887127. doi: 10.3389/fneur.2026.1887127 (PMC13572263; doi:10.3389/fneur.2026.1887127)
Supplement: Supplementary file 1 [file Supplementary_file_1.docx]

Experimental protocol of metabolic profiling analysis for serum

Samle Preparation

The 100µL sample was mixed thoroughly with 400µL cold methanolic acetonitrile (v/v, 1:1) by vortexing. They were then sonicated for 1 h in an ice bath. The mixture was then incubated for 1 h at -20 ° C and centrifuged at 14,000 g for 20 min at 4 ° C. The supernatant was then collected and dried under vacuum LC-MS analysis.

UHPLC-MS/MS analysis

Metabolomics analysis was performed using an UPLC-ESI-Q-Orbitrap-MS system (UHPLC, Shimadzu Nexera X2 LC-30AD, Japan) in combination with Orbitrap Fusion (Thermo Scientific, SAN Jose, USA). For liquid chromatography (LC) separation, samples were analyzed using an ACQUITY UPLC®HSS T3 column (2.1×100 mm, 1.8μm) (Waters, Milford, MA, USA). Mobile phase A: 0.1% formaldehyde aqueous solution, B: 100% acetonitrile (ACN), the flow rate was 0.3 mL/min. The gradient was 0% buffer B for 2 min, linearly increased to 48% in 4 min, increased to 100% in 4 min, maintained for 2 min, and decreased to 0% buffer B in 0.1 min, with a reequilibration period of 3 min.

Mass spectrometry data were collected by positive and negative modes of electrospray ionization (ESI). The electrospray source conditions were set as: spray voltage 3.8kv (positive), 3.2kv (negative), and capillary temperature 320℃. Intrathecal gas (nitrogen) flow rate: 30 arb (relative unit); Auxiliary gas flow: 5 arb; Probe heater temperature: 350℃; S-Lens RF level: 50. The instrument was set to perform a full mass spectrum scan in the m/z range from 70 to 1050 Da. Full mass spectrum scans were performed at a resolution of 70,000 at m/z 200 and 17,500 at m/z 200. The maximum injection time was set to ms 100 ms and ms/ms 50 ms. The isolation window of MS2 was set as 2 m/z, and the standardized collision energy (stepwise) was 20, 30, and 40, respectively.

Data Preprocessing and filtering

Raw MS data were used for peak alignment, retention time correction, and peak area extraction using MS-DIAL. Metabolites were identified using accuracy mass (mass tolerance < 10 ppm) and MS/MS data (mass tolerance < 0.02Da) by matching with public databases such as HMDB and massbank and a self-built metabolite standard library. Among the extracted features, only variables with more than 50% nonzero measurements in at least one group were retained.

Multivariate statistical analysis

R (version: 4.0.3) and the R package were used for all multivariate data analysis and modeling. The Pareto proportion method was used for mean-centered analysis of the data. The model was established based on principal component analysis (PCA), orthogonal partial least squares discriminant analysis (PLS-DA) and partial least squares discriminant analysis (OPLS-DA). All models evaluated were tested for overfitting using the permutation test method. The descriptive performance of the model was determined by R2X (cumulative) (perfect model: R2X (cum) = 1) and R2Y (cumulative) (perfect model: R2Y (cum) = 1) values, and the predictive performance was measured by Q2 (cumulative) (perfect model: Q2 (cum) = 1) and permutation test (n = 200). The permutation model does not predict the category: the R2 and Q2 values at the y-intercept must be lower than those of the nonpermutation model. OPLS-DA allows the use of variable projected importance (VIP) to determine the differential metabolites. The VIP score value represents the contribution of a variable to the discrimination of all category samples. Mathematically, these scores were calculated as the sum of squares of the PLS weights for each variable. The mean VIP value was 1, and usually a VIP value greater than 1 was considered significant. Higher scores are consistent with greater discrimination and thus constitute criteria for biomarker selection. At the univariate analysis level, the threshold for statistical significance of variable influence projection (VIP) values obtained in the OPLS-DA model and a two-tailed Student's t-test (p value) for standardized raw data were used to obtain the discriminating metabolites. One-way analysis of variance (ANOVA) was used to calculate p values for multi-group analysis. Metabolites with VIP values greater than 1.0 and p values less than 0.05 were considered statistically significant. The fold change was calculated as the log of the average mass response (area) ratio between any two categories. On the other hand, cluster analysis of the identified differential metabolites was performed using the R package.

KEGG Enrichment analysis

To identify the perturbed biological pathways, the differential metabolite data were performed KEGG pathway analysis using KEGG database (http://www.kegg.jp). KEGG enrichment analyses were carried out with the hypergoemetric test, and FDR correction for multiple testing was performed. Enriched KEGG pathways were nominally statistically significant at the p<0.05 level.
